# Complexities of health and care worker migration pathways and corresponding international reporting requirements

**DOI:** 10.1186/s12960-022-00780-7

**Published:** 2023-01-20

**Authors:** Ivy L. Bourgeault, Denise L. Spitzer, Margaret Walton-Roberts

**Affiliations:** 1 School of Sociological and Anthropological Studies, University of Ottawa, Ottawa, Canada; 2 School of Public Health, University of Alberta, Edmonton, Canada; 3 Geography and Environmental Studies and Balsillie School of International Affairs, Wilfrid Laurier University, Waterloo, Canada

**Keywords:** Health and care worker migration, International reporting requirements, Complexity of migration pathways, Informed policy responses

## Abstract

The increasing complexity of the migration pathways of health and care workers is a critical consideration in the reporting requirements of international agreements designed to address their impacts. There are inherent challenges across these different agreements including reporting functions that are misaligned across different data collection tools, variable capacity of country respondents, and a lack of transparency or accountability in the reporting process. Moreover, reporting processes often neglect to recognize the broader intersectional gendered and racialized political economy of health and care worker migration. We argue for a more coordinated approach to the various international reporting requirements and processes that involve building capacity within countries to report on their domestic situation in response to these codes and conventions, and internationally to make such reporting result in more than simply the sum of their responses, but to reflect cross-national and transnational interactions and relationships. These strategies would better enable policy interventions along migration pathways that would more accurately recognize the growing complexity of health worker migration leading to more effective responses to mitigate its negative effects for migrants, source, destination, and transit countries. While recognizing the multiple layers of complexity, we nevertheless reaffirm the fact that countries still have an ethical responsibility to undertake health workforce planning in their countries that does not overly rely on the recruitment of migrant health and care workers.

## Introduction

The international year of health and care workers and passing a milestone of the 10th anniversary of the WHO Code of Practice on the International Recruitment of Health Personnel (*hereafter referred simply as the WHO Code)* causes us to pause and reflect on the changing dynamics of the migration pathways of health and care workers and the international reporting requirements designed to address their impacts. It is also timely to review whether countries are keeping up to date on the targets of the 2030 Global Health Workforce Strategy, which highlights the importance of the collection of accurate health and care personnel data. In this commentary, we address two layers of complexity: first, of the migration pathways of health and care workers, and second, of the reporting on these migration pathways by countries in response to different policies, conventions, and codes. The shortages of health workers exacerbated by the COVID-19 pandemic, which initially paused but more recently accelerated health and care worker migration, makes this series of reflections and recommendations timely.

While our paper examines the multiple layers of complexity of health and care worker migration and integration that need to be captured in data collection and reporting, we maintain that countries still have an ethical responsibility to undertake robust health workforce planning in their countries so that it does not have to overly rely on the recruitment of migrant health and care workers. Further, we argue that there is a need for a more coordinated approach to the various international reporting requirements and processes that involves building needed capacity within countries to report on their domestic situation in response to these codes and conventions, and internationally to make such reporting result in more than simply the sum of their responses. This would better enable policy interventions along migration pathways that better recognize the growing complexity of health and care worker migration to help mitigate negative effects for migrants, source, destination, and transit countries.

## The growing complexity of the health and care worker migration pathway

**Fig. 1:**
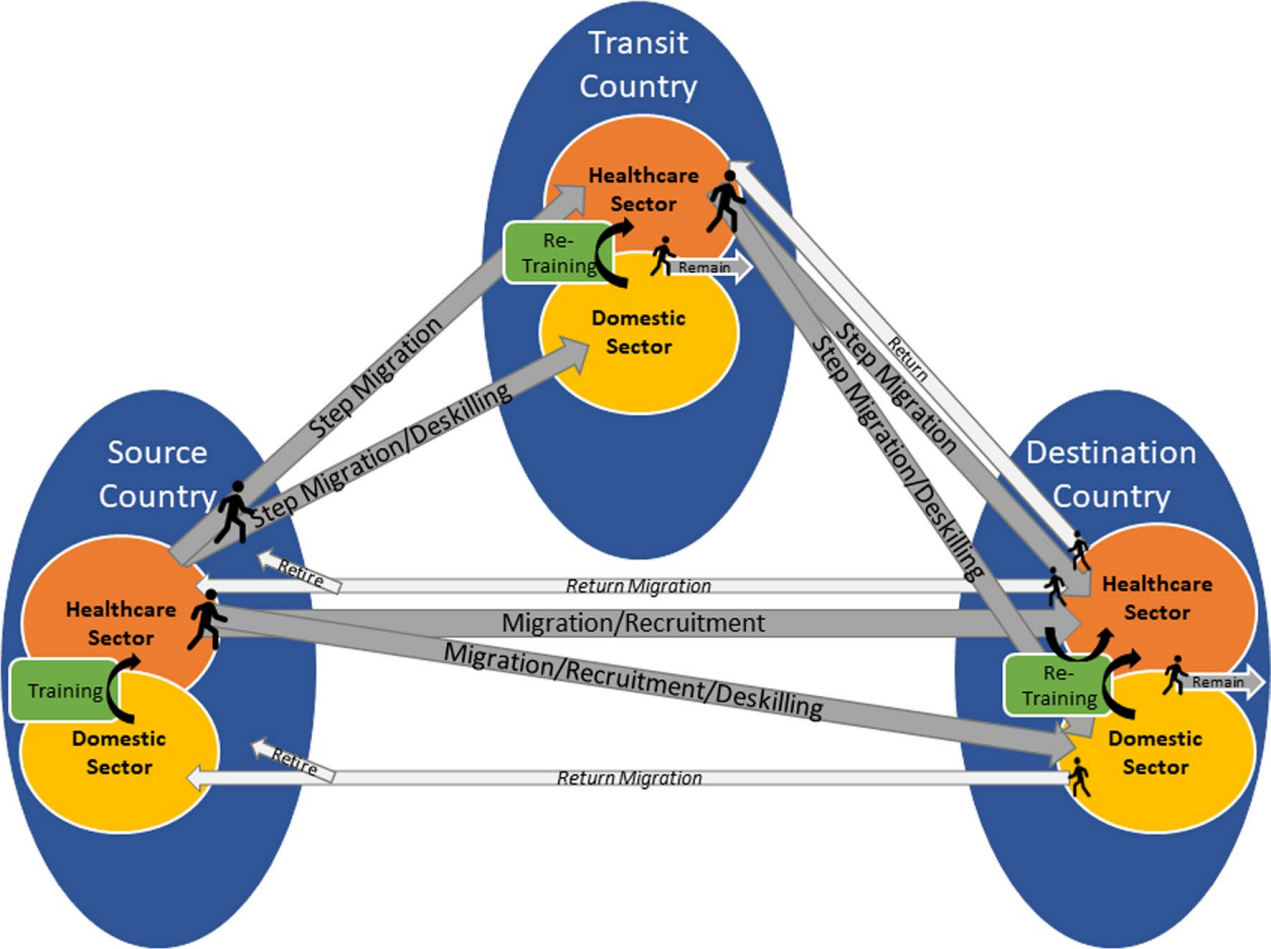
Growing complexity of migration and training pathways of health and care workers

The growing complexity of the migration pathway is a critical consideration in the reporting requirements of international agreements pertaining to the migration of health personnel. Inspired by Diallo’s [1] depiction of migration pathways, Fig. 1 outlines how the migrating health and care workers start with their countries of origin, from which they may potentially move through one (or more) transit countries to train and/or work (i.e.,* step migration*), to then arrive at an ultimate destination country.

Along these pathways, migrant health and care workers may start off or be integrated within the health sector directly; however, migrant care workers may work in the domestic sector. While some migrant care workers remain in the informal (domestic) sector, those who can transition to the health sector may require additional bridge training. Not every migrant health worker goes through all parts of the pathway depicted; some pathways are dominant, whereas others are less so (reflected partially in the size of the arrows). At different points in their careers—including retirement—they may face repatriation, engage in return migration for short or long periods of time, or depending on the policies in destination countries and the type of migrant care worker, they may remain in their destination country.

This visualization recognizes the phenomenon of two-step migration which involves migrating health and care workers’ transit through certain countries on their way to their ultimate destination [2], as well as the transition between different visa statuses within the same country, such as international student or caregiver to permanent resident pathways [3, 4]. There is additional complexity when you add in the training pathways, recognizing the growing internationalization and privatization of health and care worker education and training, during the pre and post migration phases.

The figure begins to capture the complexity of different types of workers where occupations exist along a *continuum of care* in terms of tasks and competencies, which in turn reflect a gendered and intersectional nature of care work often described as *care chains* [5–7], and how career trajectories can intersect with the opportunities and barriers presented by migration. Who is defined and included as a health and care worker also matters. Indeed, we need a better understanding of the fluidity of how these different cadres or workers intersect along migration pathways. Being inclusive of populations of interest at the outset brings into perspective a broader range of international agreements and reporting requirements that may otherwise be siloed.

Migrant domestic workers, regardless of educational background, are vital to our considerations of health and care worker migration as their role in maintaining and promoting household health can produce a proportional loss of care resources in source countries. This loss is intensified by the de-skilling of trained health professionals who are recruited as migrant care workers in private households. The legacies of colonialism, neo-colonialism, and neoliberal globalization, which reinforce gender ideologies that assign and devalue care work as the ‘natural’ domain of women, has contributed to the paucity of remunerative care labour in countries of the global South; this, along with the promotion of migration as a tool of development, has normalized out-migration of health and care workers even into roles in which they are deskilled [8, 9]. The low value often placed on care work also informs this migration cycle contributing to the precarity faced by migrant care workers along these pathways [4, 10]. For example, Filipina health and care workers who migrate under the auspices of migrant care worker schemes, such as Canada’s Caregiver Program, formerly as the Live-In Caregiver Program, in the hopes of re-claiming their prior status as health professionals are often stalled in the process. The length of time required to transition from temporary foreign worker to permanent resident through one of the designated pathways generally runs out the clock on their credential recognition, this requires them to undertake further education and outlay of finances, which they can ill-afford given the need and demand for remittances to be sent home [11].

We recognize that other complexities at the level of *health and migration systems* are not fully captured in this figure. For example, although recruitment is depicted, the role of public and private sector *intermediaries* along the migration pathway are invisible. It is also important to note that there is not a clear distinction between source, transit, and destination countries—countries can have both incoming and outgoing health workers. It is also difficult to capture who shares in the benefits of migration in the context of rapidly privatized training and education, where it is difficult to determine who is ultimately paying for and benefiting from these investments.

Other complexities at the level of *migrating health and care workers* that are difficult to visually depict include the complexity across the life course, especially regarding transnational familial connections. Also, although the figure depicts the role of training along the pathway, it does not capture the important distinction between migrating health and care workers who are both born and trained outside of a transit or destination country, those who are born internationally but domestically trained (in some cases as international students), and those who are domestically born but internationally trained (*c.f.,* [12].

## The resulting complexity of the reporting across international agreements, codes and conventions

**Fig. 2:**
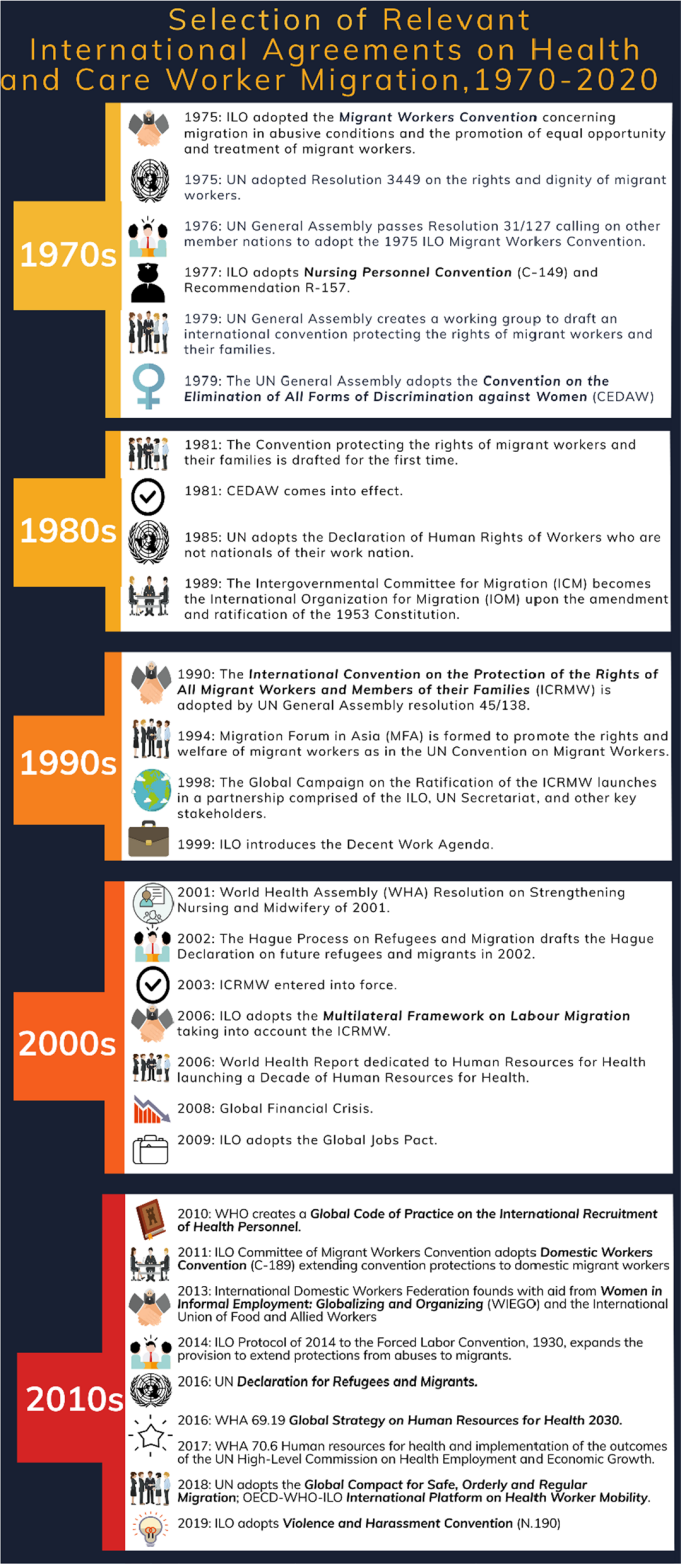
Selection of relevant international agreements on health and care worker migration, 1970–2020

The WHO Code and the reporting requirements that it encompasses is but one tool in the protection of the rights of migrating health and care workers as well as health and care systems and by extension the rights of non-migrating health and care workers. Indeed, there are several international agreements, codes and conventions that impact the different types of migration pathways of health and care workers across different country contexts, some of which are listed in chronological order of development in Fig. 2. Each of these agreements, codes and conventions are historically situated reflecting the complexity of health and care worker migration of the era when they were developed. The impact of these different agreements, codes and conventions is dependent on countries’ commitments to them, existence of local policies and programmes developed in concordance, and the completeness of the cyclical reporting on these activities.

For discussion, we have included examples below (in alphabetical order). These are not meant to be an exhaustive list, but make the point of the complexity that emerges when we consider these international instruments in total. We note how for each institution overseeing these agreements, codes, and conventions there are different reporting standards. Moreover, we recognize that political and bureaucratic factors shape the ability and interest of states to report and comply with requirements [13].

### International Labour Organization (ILO)

As noted in Fig. 2, there are two ILO Conventions pertaining to the migration of health and care workers including C149 Nursing Personnel Convention, 1977 and C189 Domestic Workers Convention, 2011. As technical conventions, their standards *benchmark ‘*decent work in all its dimensions and aspects’, including formalization of work and worker protection. Components of these conventions and recommendations acknowledge actual or potential differences in the status and treatment of *migrant* nurses and *migrant* domestic workers. They attach legal obligations to ratifying countries and guide and inform policy at different governance levels. Countries are encouraged to incorporate Convention standards in their health policy frameworks and provide policy guidance for international cooperation, such as bilateral agreements that oblige signatories to the actions, principles of cooperation and goals detailed in memoranda of understanding (MOUs) [14].

The reporting requirements for the ILO are reviewed by their Committee of Experts on the Application of Conventions and Recommendations (CEACR), composed of 20 legal scholars from around the world [15]. If a country ratifies a Convention, it is then responsible for reporting on its implementation every three years regarding fundamental and governance agreements. Reporting for all other conventions occurs every six years. Reports are to be shared by governments with worker and employer organizations to enable these stakeholders to provide supplementary comments directly to the ILO regarding implementation.^1^ The CEACR may offer observations about the country’s application of the Convention, which are made public in their annual report, and may make requests for further information. This process facilitates conversation amongst governments, the ILO, civil society, and private actors [15].

### United Nations (UN)

As noted in Fig. 2, the International Convention on the Protection of the Rights of All Migrant Workers and Members of their Families (ICRMW) adopted in 1990 and the Global Compact for Safe, Orderly and Regular Migration adopted in 2018 came into effect through a campaign involving numerous UN agencies and the IOM (which became a related organization of the UN in 2016).^2^ First, states ratifying the ICRMW must report on their efforts to give effect to the Convention within 1 year after the Convention comes into force for that country, and thereafter every 5 years.^3^ As of December 2021, the ICRMW has only been ratified by 56 countries, and the majority of these are predominantly migrant sending states.^4^ Second, the Global Compact reporting structure is through an intergovernmental global platform ‘the International Migration Review Forum’, where Member States discuss their progress in implementing the Global Compact. These forums are intended to occur every four years and include all stakeholders. The Global Compact is also explicit about its relationship to goals included in the 2030 Agenda for Sustainable Development.

Complementing these instruments, the UN High-Level Commission on Health Employment and Economic Growth released its report in 2016. It issued a five-year action plan 2016–2021 aimed at expanding a sustainable health workforce that promotes healthy lives, inclusive growth and equity and economic security for all [16]. The SDGs include agreements on the global indicator framework which includes 231 unique indicators. These are to be refined annually, and reviewed by the UN Statistical Commission in 2020 and 2025.^5^ These indicators include the proportion of total government spending on essential services (education, health and social protection) under 1.a 2., and health worker density and distribution under 3 c.1., and 8.8.2 level of national compliance with labour rights (freedom of association and collective bargaining) based on International Labour Organization (ILO) textual sources and national legislation, by sex and migrant status under 8.8.2.^6^

### World Health Organization

Finally, the key WHO instrument relevant to the migration of health and care workers is its Global Code of Practice on the International Recruitment of Health Personnel adopted in 2010. Global health diplomacy has been effective in the creation of a series of voluntary codes of conduct to discourage health worker migrant recruitment from countries experiencing crisis level shortfalls in medical staffing. The WHO Code, adopted in May 2010, is considered a landmark agreement that “suggests evolution in the capacity of the WHO Secretariat, Member States, and civil society to engage in global health ‘law-making’” [17]. One of the main motivations for voluntary codes is a desire for transnational social justice, because the ‘brain drain’ of health workers represents an inequitable distribution of training investments made by the sending region. The goal of the WHO code is to reduce the active recruitment of health workers from countries facing critical shortages of health workers, and for high income receiving nations to commit to achieving health worker self-sufficiency. The reality of achieving this, or of even assessing where countries stand in terms of the relative supply of health care workers through domestic training versus immigration, is challenging to assess. Nevertheless, the WHO Code encourages member states to submit reports every three years on measures taken to implement the Code. Reports from all relevant stakeholders on activities related to the implementation of the Code are considered.^7^

### Challenges and limitations

There are several inherent challenges across these different agreements, codes, and conventions. They include aspirational and generalized goals, whereas stakeholders (unions, employers, migrants, governments) have very specific needs and interests that may contradict certain aspects of the voluntary codes. They normally only apply to large public institutions or government health sectors, and thus are limited in coverage making them difficult to enforce [18, 19]. Additionally, many of these reporting functions are not fully aligned across different tools to collect data from countries, different levels of tacit and codified knowledge, varied bureaucratic capacity of individual country respondents to complete these reports [13], as well as a lack of transparency in the reporting process. Reporting structures and processes can also fail to capture data reflecting the broader intersectional gendered and racialized political economy of health and care worker migration. As Wickramasekara [20] suggests, there remains a gap between the promise and delivery of such agreements. In the case of health and care worker migration, we argue that the consequences of an uncoordinated approach are specific and material in terms of health and migration systems at the macro-level as well as at the micro-level experiences of individual health and care workers.

#### Reporting requirements

As noted above, each of these different international agreements have different reporting requirements with different processes, timing, and target information. Indeed, part of the problem is the sheer number of international agreements relevant to the international migration of health and care workers, and how the reporting for each of these might not generate the outcomes desired of any of them. At the global level, these agreements, codes, and conventions are meant to articulate with each other and other policies, but if this is not built into the reporting mechanisms this may be an unrealistic goal. How do these agreements, codes, and conventions work in tandem? When new agreements, codes, and conventions are adopted or ratified, it may not be clear how these articulate/crosswalk with other agreements. Does one feed into the next? Do new targets take over or subsume previous targets? How do these get rolled into the next agreement, code, and convention? But more to the point of this commentary, what is the impact on reporting? When targets change, it can be difficult to fully assess impact.

#### Data collection tools

Robust reporting processes for desired targets require high-quality data collection methods (i.e., survey instruments) and the right respondents with access to the requisite knowledge. Reporting on international agreements, codes and conventions can suffer from these two limitations.

In terms of *content*, survey instruments should ensure coverage of key topics. An explicit intersectional lens can be lacking in the collection, reporting and synthesis of the data provided by countries; this can illuminate the heterogeneity of migrant health and care workers, and how their social positioning may be altered through migration and integration processes. Historic and contemporary specificities inform the construction of the labour market in destination and transit countries and the subsequent reception and placement of these gendered and racialized workers. There can be inherent data and evidence limitations within countries in this regard; for example, from where we are writing in Canada there has been little intersectional data available beyond gender as a binary given our lack of race-based data.

In terms of *form*, survey instruments should limit overlap across questions and verify that each question addresses only one dimension or topic. Technical assistance to ensure the best form of questions to elicit content is a leading practice in the design of survey instruments. There is value in standardized or pre-tested questions with validated measures across reporting surveys where there is overlap in terms of content. There should be clarity on the measures or granularity of data requested—either at a high level or more granular reflecting the interplay between policies and lived experiences of migrating health and care workers. Surveys should also be designed to ensure ease of completion by participants—digitally enabled with some pre-determined drop-down menus or pre-populated from previous responses to ensure greater completeness and linkage longitudinally. Responses can also be more inclusive of all stakeholders (government, employer, labour and migrant worker representatives).

#### Capacity of country respondents

With the confluence of various agreements, codes and conventions, our quest for more (and better) data may overwhelm the capacity of institutions to provide it; that is, respondent burden is an important issue to recognize especially in cases where bureaucratic capability is limited [13]. It can be difficult to determine which Ministry should be providing country-based responses to reporting surveys (e.g., health or labour or immigration or trade), which level of government in the case of federated systems, which people with the required knowledge within or across Ministries and what capacity each has to respond to the surveys. If more than one department is involved, it is unclear whether respondents are (or should be) in conversation with one another, raising the persistent challenge of the lack of communication and policy action across government departments. The issue of who prepares country reports, their competency and access to knowledge and data can also be a limiting factor. Across reporting periods, it is unclear whether there is institutional memory given the rapid turnover of civil servants within and across reporting departments.

Corresponding mention of relevant agreements between national partners is limited (such as bilateral labour or mutual recognition agreements). It is unclear internationally what processes are in place to link responses between countries, especially those with existing bilateral and multilateral agreements, and what capacity exists or is needed within countries and/or the international organizations who manage the reporting process to assess such linkages to ensure reliability and validity of the survey responses. The ability to crosswalk data between countries would need to be built into the data collection process, creating datasets with greater reliability and higher quality.

#### Lack of transparency in the reporting process

Finally, there can be a lack of transparency in the reporting process and accountability for the data provided (or not provided). It is not clear whether there is any recourse for countries for not responding to surveys in their entirety, to specific questions, or for confirming relevance, specificity, reliability, and validity of responses. Added to this, there is often a lack of longitudinal linkages between reports across reporting periods. Capacity for these data quality features requires explicit attention.

## Promising reporting practices

There are some promising practices that if implemented could potentially improve the current somewhat uncoordinated and diffuse reporting structures, processes and outcomes across different agreements, codes, and conventions.

There is a need to improve the data collection tools and processes balancing the need to change content to reflect emerging dynamics without compromising the ability to see trends over time (e.g., using standardized elements within and across reporting processes), and how there may be a roll-over of targets included in the new agreements. Data pertaining to the two- (or more) step pathways migrant health and care workers undertake as international students and on skill utilization of migrant workers are particularly needed. Data capturing the intersectional nature of health and care worker migration and impacts on health and migration systems are also critical. Questions typically address the present circumstances but could be augmented with reference to past accomplishments as well as future aspirations in line with the goals of different agreements, codes, and conventions to add a longitudinal dimension to the reporting process.

A promising strategy to improve the reporting process is for it to be made more open and transparent so that data provided, and the syntheses and analyses taken thereof, are of higher quality. Greater utilization of Technical Expert Groups (TEG) with the required expertise not only in the breadth of health and care worker migration experiences at the individual and system levels in source, transit, and destination countries, but also methodological expertise in international survey design and support are to be encouraged. Pre-testing of the content and form of data collection tools and platforms with input from experts in academia, civil society (including migrant advocacy and migrant worker organizations) and ministry policymakers/country respondents would help to make for more robust reporting processes and outcomes.

Attention to the resources needed within and across countries internationally for high-quality data collection would yield higher quality syntheses and analyses. Partnering across source and destination countries, especially those with bilateral agreements, could enable the sharing of resources for stronger, coordinated and more complete responses. There would be an added benefit of knowledge shared across bilateral partners. Similarly, partnering between established and ‘new’ ministry respondents within and across countries could yield more accurate and complete responses. ‘Rewards’ of some kind for accuracy and completeness of responses could be considered, perhaps in the form of more resources for reporting in the next round or recognition through membership on the TEG.

Other forms of triangulation to improve reliability and validity of the data being reported should be considered, akin to an open peer review process. More insights can be provided when other groups provide data and review draft responses. Regular engagement with migrant worker organizations is critical to both the amplification of their voices and concerns with regard to the implementation and reporting on codes and conventions and to the ongoing education of members as to their rights and protections under these agreements. In this regard, it is excellent to see that in the latest reporting on the WHO Code, responses from academia and civil society organizations (many of whom have longer standing interest in and expertise on the topic than some country respondents) were particularly encouraged, which we anticipate will be an effective way of crowdsourcing data for more complete responses. Similarly, as noted above, the reporting process for the ILO conventions includes an obligation to consult and share the initial report with civil society organizations and report back to the ILO. This approach can foster greater social dialogue on key issues, expose emerging issues of concern, and is worthy of greater promotion with each round of reporting.

Any opportunity for alignment and coordination through institutional linkages across organizations and country responses within and between countries are to be encouraged. An online platform where country responses to relevant reports are gathered/curated in one place would be ideal. In this regard, we are encouraged by the ongoing collaboration between the Organization for Economic Cooperation and Development with the WHO and ILO on the International Platform on Health Worker Mobility established in 2018. Such partnerships and joined up policy at the international level with clear impacts at the country level should be accelerated especially in the ‘build back better’ post COVID-19 phase.

## Conclusions

The internationalized nature of the health and care workforce and the continued role that immigration plays in addressing workforce shortages necessitates that we understand the complexity of the migration cycle, the range of agreements, codes and conventions that relate to the migration of such workers, and the problems associated with this reporting landscape to generate better policies and practices.

Additionally, there is a need for greater specificity regarding intersections of migration, gender, and value of care work, a timely issue to address as the importance of care work across the continuum has become more salient in the context of the COVID-19 pandemic. This pandemic has also raised the issue of who counts as health and care workers. In many countries, domestic care workers still fall outside of labour codes and protections, while they have borne increasing responsibilities under conditions of the pandemic. Migrant care workers who are or become undocumented because of pandemic policies or pauses face even greater precarity in the course of their lives and erasure from the official reporting that is the topic of this commentary.

As we cautioned at the outset, in reviewing the complexity of health worker international migration and the related reporting instruments, we do not want to lose sight of one of the core issues the WHO Code raises, that is the ethical obligations that states must adhere to regarding the global distribution of health workers, namely that countries must invest in their own health and care workforce capacities. This is especially critical considering the enormous stresses faced by these workforces as a result of the pandemic.

We also do not want to lose sight of the humanity of individuals living and working along these migration pathways and the impact on themselves, their families and communities. International agreements, codes and conventions are vital tools in promoting the international obligation to support the Decent Work and Sustainable Development agendas in relation to the international migration of health and care workers. The COVID-19 pandemic has revealed the need for continued improvement in the relevance and reporting contexts for these instruments.

## Data Availability

Not applicable.

## Abbreviations

CEACR: Committee of Experts on the Application of Conventions and Recommendations (ILO)
CEDAW: Convention on the Elimination of All Forms of Discrimination against Women
ICRMW: International Convention on the Protection of the Rights of All Migrant Workers and Members of their Families
ILO: International Labour Organization
IOM: International Organization on Migration
MFLM: Multilateral Framework on Labour Migration
MOU: Memorandum of Understanding
OECD: Organization of Economic Development and Cooperation
TEG: Technical Expert Group(s)
UN: United Nations
WHO: World Health Organization
WIEGO: Women in Informal Employment: Globalizing and Organizing

## Footnotes

1 https://www.ilo.org/global/standards/applying-and-promoting-international-labour-standards/committee-of-experts-on-the-application-of-conventions-and-recommendations/lang--en/index.htm Accessed December 28 2021.

2 Global Compact For Safe, Orderly and Regular Migration Intergovernmentally Negotiated and Agreed Outcome https://refugeesmigrants.un.org/sites/default/files/180713_agreed_outcome_global_compact_for_migration.pdf Accessed December 28 2021.

3 Article 73 https://www.ohchr.org/en/professionalinterest/pages/cmw.aspx Accessed December 28 2021.

4 https://indicators.ohchr.org/ Accessed December 28 2021.

5 https://unstats.un.org/sdgs/indicators/indicators-list/ Accessed December 28 2021.

6 https://unstats.un.org/sdgs/indicators/Global%20Indicator%20Framework%20after%202021%20refinement_Eng.pdf Accessed December 28 2021.

7 https://www.who.int/teams/health-workforce/migration/code-nri/ Accessed December 28 2021.
